# Regulatory mechanisms of exopolysaccharide synthesis and biofilm formation in Streptococcus mutans

**DOI:** 10.1080/20002297.2023.2225257

**Published:** 2023-06-18

**Authors:** Ting Zheng, Meiling Jing, Tao Gong, Jiangchuan Yan, Xiaowan Wang, Mai Xu, Xuedong Zhou, Jumei Zeng, Yuqing Li

**Affiliations:** a State Key Laboratory of Oral Diseases, National Clinical Research Center for Oral Diseases, West China Hospital of Stomatology, Sichuan University, Chengdu, China; b Department of Operative Dentistry and Endodontics, West China Hospital of Stomatology, Sichuan University, Chengdu, China; c West China School of Public Health and West China Fourth Hospital, Sichuan University, Chengdu, China

**Keywords:** Dental caries, *streptococcus mutans*, biofilm formation, exopolysaccharides synthesis, regulatory mechanism

## Abstract

**Background:**

Dental caries is a chronic, multifactorial and biofilm-mediated oral bacterial infection affecting almost every age group and every geographical region. *Streptococcus mutans* is considered an important pathogen responsible for the initiation and development of dental caries. It produces exopolysaccharides *in situ* to promote the colonization of cariogenic bacteria and coordinate dental biofilm development.

**Objective:**

The understanding of the regulatory mechanism of *S. mutans* biofilm formation can provide a theoretical basis for the prevention and treatment of caries.

**Design:**

At present, an increasing number of studies have identified many regulatory systems in *S. mutans* that regulate biofilm formation, including second messengers (e.g. c-di-AMP, Ap4A), transcription factors (e.g. EpsR, RcrR, StsR, AhrC, FruR), two-component systems (e.g. CovR, VicR), small RNA (including sRNA0426, srn92532, and srn133489), acetylation modifications (e.g. ActG), CRISPR-associated proteins (e.g. Cas3), PTS systems (e.g. EIIAB), quorum-sensing signaling system (e.g. LuxS), enzymes (including Dex, YidC, CopZ, EzrA, lmrB, SprV, RecA, PdxR, MurI) and small-molecule metabolites.

**Results:**

This review summarizes the recent progress in the molecular regulatory mechanisms of exopolysaccharides synthesis and biofilm formation in *S. mutans*.

## Introduction

Dental caries is a common chronic infectious disease caused by bacteria that seriously endangers the oral and systemic health [1,2]. According to the Global Burden of Disease (GBD) 2019 study, the caries rate of permanent teeth ranks first among 369 major diseases [3]. An estimated 2.0 billion (95% uncertainty interval, 1.8 to 2.3 billion) people with untreated caries of permanent teeth are present worldwide, and an age-standardized prevalence of 25,625.5 (22,281.1 to 29,372.0) per 100,000 person/year in 2019 [4,5]. Additionally, caries in deciduous teeth ranks first among all disorders in children aged 0 to 14 years in 2019, with 0.5 billion (0.4 to 1.6 billion) cases of caries in deciduous teeth and an age-standardized prevalence of 7,672.9 (6,113.1 to 9,339.6) [4,5]. *S. mutans* is closely related to the initiation and development of caries due to its aciduric and acidic properties, and ability to synthetize glucan to form biofilm [6]. The ability to synthesize large amounts of glucan to form biofilm contributes to the permanent colonization of tooth surfaces by *S. mutans* and the local development of the extracellular polymer matrix [7].

*S. mutans* biofilm formation is regulated by two independent mechanisms: sucrose-dependent and sucrose-independent [8,9]. In the absence of sucrose or early stages of adhesion, the interaction of antigen I/II (also known as P1, SpaP, Ag I/II, or PAc) with agglutinins in the saliva mediates the adhesion of *S. mutans* to the dental surfaces and bacterial aggregation [7,10–12]. In the presence of sucrose, glucosyltransferases secreted by *S. mutans*, including GtfB, GtfC and GtfD, utilizes the glucose part of sucrose as a substrate to form the growing polymer of glucan, which is also called exopolysaccharides [7,13]. The presence of exopolysaccharides enhances the strength of the local adhesion of *S. mutans* to the tooth surfaces [14]. Moreover, the continuous production of exopolysaccharides *in situ* further expanded the three-dimensional matrix while forming a bacterial cell core wrapped by exopolysaccharides [15]. It also provides a scaffold for the colonization of other microorganisms (e.g. *Candida albicans*) and the adhesion of other substances (including eDNA, lipoteichoic acids, proteins), further leading to biofilm formation [16].

**Figure 1.**
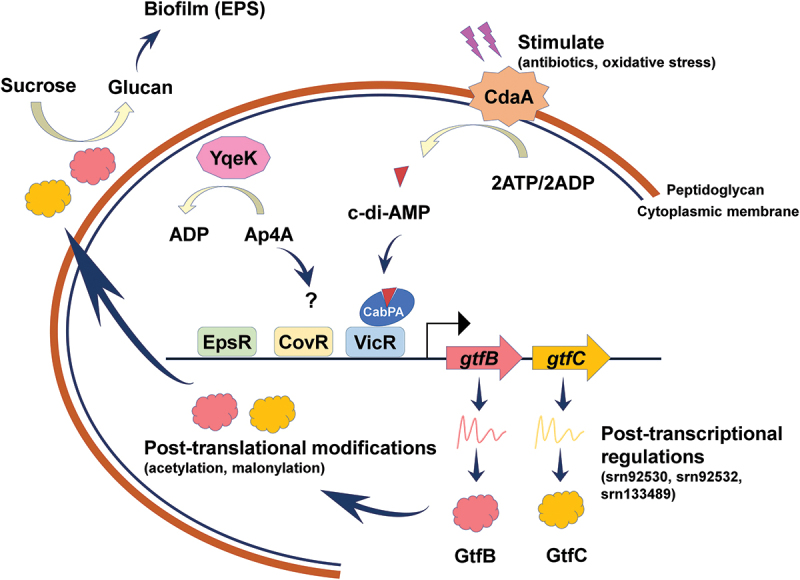
The regulatory mechanisms of exopolysaccharides synthesis and biofilm formation in *S.* *mutans*. c-di-AMP controls *S.* *mutans* biofilm formation by regulating *gtfB* expression through the binding of CabPA to VicR, while Ap4A represses the expression of *gtf*s through some unknown pathways. VicR, EpsR and CovR regulate *S.* *mutans* exopolysaccharides synthesis and biofilm formation directly by binding to the promoters of *gtfB* and *gtfC*. Post-translational modifications are also involved in the regulation of Gtfs activities and biofilm formation in *S.* *mutans*.

This review summarizes the molecular regulatory mechanisms affecting the formation of *S. mutans* biofilm. The signals that regulate the formation of *S. mutans* biofilm are considered from five different aspects: (1) upstream signals, such as second messengers; (2) transcriptional level, such as transcription factors and two-component systems; (3) post-transcriptional level, such as small RNAs; (4) post-translational level, such as acetylation, malonylation; (5) others, such as CRISPR-related proteins, PTS systems, quorum-sensing signaling system, enzymes and small-molecule metabolites (Figure 1).

## Regulatory formation of *S. mutans* biofilm from upstream signals

### Second messengers

Bacteria use second messengers to conduct signals in response to the rapid change of the environment. In recent years, many nucleotide molecules or second messengers have been found as regulators of various physiological activities of bacteria, including biofilm formation [17].

#### c-di-AMP

c-di-AMP is an important and ubiquitous second messenger in bacterial signaling, whose intracellular levels are regulated by the enzymes diadenylate cyclase (DAC) and phosphodiesterase (PDE) [18]. DAC contains a highly conserved DAC/DisA_N domain, which utilizes two molecules of ATP or ADP as a substrate to synthesize c-di-AMP [19,20]. PDE contains a DHH/DHHA1 (Asp-His-His)/HD (Asp-His) domain, which breaks down c-di-AMP into two AMP molecules or 5′-phosphoadenylyl-(3′−5′)-adenosine (5’-pApA) [21,22]. Therefore, the DAC and PDE processes keep the intracellular c-di-AMP level within an appropriate range.

The DAC and PDE enzymes in *S. mutans* are encoded by *cdaA* and *pdeA,* respectively. Cheng *et al*. found that the c-di-AMP levels of *S. mutans* decreased significantly after knockout *cdaA* [23]. Genes with increased expression were clustered in cellular polysaccharide biosynthetic processes, including *gtfB*, *gtfC* [23]. Besides, both GTF enzyme activity and their expression at the gene level were significantly increased. Nevertheless, Peng *et al*. constructed a similar in-frame deletion mutant obtaining different results [24]. Indeed, DAC deficiency significantly reduces the expression of *gtfB* and inhibits the synthesis of exopolysaccharides, but does not markedly affect the expression of *gtfC* and *gtfD*. Therefore, the involvement of c-di-AMP in the formation of *S. mutans* biofilms occurs through the regulation of gtfB expression. In addition, Peng *et al*. revealed that CabpA and CabpB are two receptor proteins for c-di-AMP [25]. CabPA interacts with VicR, a response regulator that regulates *gtfB* expression, thereby promoting biofilm formation. These results demonstrate that c-di-AMP is an upstream signal that regulates biofilm formation in *S. mutans.*

#### Ap4A

Ap4A is a dinucleotide metabolite that consists of two adenosines joined in the 5′−5′ linkage by four phosphates [26,27]. During translation, aminoacyl-tRNA synthetases (AARS) catalyze the transfer of the AMP moiety from the aminoacyl adenylate to the ATP to form Ap4A in the absence of tRNA [28]. Ap4A is symmetrically decomposed into two molecules of ADP or asymmetrically into AMP and ATP by the Ap4A hydrolase YqeK, which was identified and characterized in *S. mutans* [29]. Zheng *et al*. showed that the in-frame deletion of the *yqeK* gene in *S. mutans* causes an increase in the intracellular Ap4A levels, while biofilm formation and water-insoluble exopolysaccharides production are decreased [30]. The knockout of the *yqeK* gene leads to the downregulation of important virulence genes related to biofilms, such as *gtfB*, *gtfC*, and *gtfD* [30]. Although the mechanisms involved in the role of Ap4A need to be further investigated, these results suggest that it affects biofilm formation in *S. mutans* by affecting the expression of *gtf*s and the activity of Gtfs.

## Regulation of *S. mutans* biofilm formation at the transcriptional level

### Transcription factors

Some transcription factors also play a very important role in the formation of *S. mutans* biofilm. The exopolysaccharides synthesis regulator (*epsR*) gene is one of the transcription factors of the multiple antibiotic resistance regulator (MarR) family in *S. mutans*. Chen *et al*. demonstrate that EpsR specifically binds to the promoter regions of *gtfB*, consequently negatively regulating *gtfB* expression and exopolysaccharides production in *S. mutans* [31].

RcrR, a *rel* competence-related regulator, is a transcription factor belonging to the multiple antibiotic resistance regulator (MarR) family in *S. mutans*. RcrR specifically binds to the promoter regions of *Smu.1185* and *Smu.2038* to regulate the carbohydrate transport of mannitol and trehalose-specific PTS system, and it also has a certain impact on the transport of several carbohydrates, including glucose, galactose, lactose, and maltose [32]. The deletion of the *rcrR* gene in *S. mutans* decreases the ability to form the biofilm and affects the expression of its multiple genes related to sugar transportation [32].

StsR is the transcription factors of GntR family that specifically binds to the predicted promoter sequences in the mannitol-specific PTS transporter, the mannose-specific PTS transporter, maltose ABC transporter and multiple sugar-binding ABC transporter in *S. mutans* [33]. When *stsR* is knocked out, the amount of biofilm and the content of exopolysaccharides of *S. mutans* decreases significantly at the early stage [33].

AhrC is a transcription factor of the arginine repressor (ArgR) family in *S. mutans*. The results of electrophoretic mobility shift assay (EMSA) and DNase I footprinting showed that AhrC specifically binds to the *argC* promoter region [34]. AhrC also binds to the promoter regions of *argG*, *Smu.815*, *Smu*.1904, and *Smu*.940c to regulate the downstream gene expression [34]. In addition, the *S. mutans* strain overexpressing *ahrC* has a significant decrease in the formation of biofilm and content of water-insoluble exopolysaccharides [34]. These results suggest that AhrC negatively regulates arginine biosynthesis and biofilm formation in *S. mutans.*

FruR is a deoxyribonucleoside repressor (deoR)-type regulator that co-transcribes a single EIIABC^Fru^ permease with FruI, and a putative 1-phosphofructokinase (1-PFK) with FruK. The loss of FruR results in a significantly reduction of *S. mutans* biofilm [35].

PdxR is encoded by *smu.864*, a member of the GntR superfamily of regulatory proteins. When *S. mutans* is grown in defined medium, *pdxR* deficiency affects its growth of and significantly reduces biofilm formation [36].

A total of 130 transcription factors were predicted in *S. mutans* (https://mistdb.com/). However, the functions of several transcription factors remain unknown. Therefore, further research is necessary to investigate the relationships between the uncharacterized transcription factors and biofilm formation.

### Two-component systems

Bacterial two-component systems are regulatory circuits of signal transduction based typically on a membrane bound sensor histidine kinase (HK) and a cytoplasmic response regulator (RR) that is activated through a histidine to aspartate phosphorelay reaction [37]. At present, 14 two-component systems and one orphan regulator (CovR) have been found in *S. mutans* [38]. Among them, VicR positively regulates the expression of *gtf*s by binding to the promoter region of *gtfB* and *gtfC* [39,40]. CovR (also known as GcrR) is a negative regulator that inhibits the expression of *gtfB* and *gtfC* by directly binding to the promoter region [41].

Zhang *et al*. constructed a series of mutant strains (including *vicR*+*covR*+, *vicR* and *covR* overexpression; *vicR*+*cov*-, *vicR* overexpression and *covR* deficiency; AS*vicRcovR*+, *vicR* low-expression and *covR* overexpression; AS*vicRcovR*−, *vicR* low-expression and *covR* deficient) to explore the synergy between VicR and CovR in *S. mutans* in the regulation of the sucrose-selective exopolysaccharides metabolism, and they found that *gtfB*/*gtfC* expression is mainly regulated by *covR* regardless of the *vicR* gene expression, as revealed by the analysis of the biofilm content of these several strains [42]. These results suggest that CovR may play a dominant role in the interaction between CovR and VicR affecting the expression of *gtf*s.

VicK is a histidine protein kinase that monitors and transmits chemical signals to the downstream regulatory proteins such as VicR and CovR in *S. mutans* [43]. Subsequently, VicR or CovR regulate biofilm-associated genes at a transcriptional level, such as *gtfB/C*, *ftf* and *gbpB* [44]. A study found that the deletion of the *vicK* gene in *S. mutans* suppresses biofilm formation as well as exopolysaccharides production, and the expression of genes related to exopolysaccharides synthesis are down-regulated, with the exception of *gtfB* [45]. In addition, the expression of *vicX* and *covR* genes are decreased after *vicK* knockout. These results indicate that VicK regulates biofilm formation by affecting CovR and VicR.

## Regulation of *S. mutans* biofilm formation at the post-transcriptional level

Small RNAs (sRNAs) in bacteria are typically 50–400 nucleotides in length, playing a key role in regulating gene expression [46,47]. In the canonical pathway regulating sRNA-mediated gene expression, sRNAs, often along with the chaperone protein Hfq, positively or negatively regulate the target mRNA expression in response to environmental changes through an incomplete Watson–Crick base pairing [48]. Some studies discovered the presence of many sRNAs with regulatory functions in *S. mutans* [49–51]. Yin *et al*. detected a potential sRNA regulation pathway in *S. mutans* by bioinformatics technology [52]. The results showed that sRNA0426 has a strong positive relationship with the dynamic biofilm formation in *S. mutans*. Furthermore, the expression of *gtfB* and *gtfC* mRNAs are positively correlated with sRNA0426 expression. Liu *et al*. evaluated the expression of sRNAs and target genes (*gtfB*, *gtfC*, and *spaP*) related to virulence, and they found that the target mRNA of srn92530 is *gtfB*, while the target mRNA of srn92532 and srn133489 is *gtfB* and *gtfC*, respectively [53].

The *rnc* gene is considered the coding gene of ribonuclease III (RNase III), which can promote mRNA maturation [54]. Mao *et al*. observed that the *rnc* gene is located upstream of the *vicRKX* tricistronic operon and the downstream *vic* locus is repressed by *rnc* at the mRNA level in *S. mutans*. Three putative microRNA-size small RNAs (msRNAs), such as msRNA1701, msRNA3405, and msRNA1657, are negatively correlated with *vicRKX* but positively correlated with *rnc*, as revealed by deep sequencing and bioinformatics analysis [55]. Moreover, they found that the knockout *rnc* gene reduces the ability of biofilm formation of *S. mutans* [56].

Antisense RNA (AS RNA) refers to an RNA that inhibits the expression of related genes after complementing with mRNA [57]. Lei *et al*. detected an AS RNA named ASvicR upstream of the *rnc* gene in *S. mutans*, whose overexpression inhibits the transcription of *vicR, gtfB, gtfC* and *gtfD*, resulting in the decrease of the exopolysaccharides synthesis and biofilm formation [58,59]. Moreover, msRNA1657, a microRNA-size small RNA, binds to the 5′-UTR region of the *vicR* gene [59,60]. This suggests that ASvicR and msRNA1657 regulate *gtf*s transcription through the *vicR* gene. At present, the detailed mechanisms of small RNA affecting the biofilm formation of *S. mutans* is still not well-known.

## Regulation of *S. mutans* biofilm formation at the post-translation level

Protein translational modifications (PTMs) increase the functional diversity of proteome through the covalent addition of functional groups or proteins, the proteolytic cleavage of regulatory subunits or the degradation of the whole protein [61,62]. PTM has important functions in regulating the physiological activities of *S. mutans*. For example, glutathionylation on the Cys41 residue of Tlp is crucial to protect *S. mutans* from oxidative stress and in the competition with *S. sanguinis* and *S. gordonii* [63]. Lei *et al*. identified 973 acetylation sites in 445 proteins of *S. mutans*, and among them, 617 acetylation sites in 302 proteins were quantified, revealing that the activity of GtfB, GtfC, and GtfD is regulated by acetylation [64]. A recent study found that the GCN5-related N-acetyltransferases (GNAT) family member ActG catalyzes the acetylation of GtfB and GtfC in *S. mutans*, subsequently inhibiting the synthesis of water-insoluble exopolysaccharides and biofilm formation [65].

Li *et al*. performed a global protein lysine malonylation (Kmal) analysis of *S. mutans* and identified a total of 392 malonyllysine sites in 159 proteins [66]. Among them, proteins (WapA, SpaP, GtfC, Ftf, GbpB) closely related to *S. mutans* biofilm formation were modified with Kmal, suggesting that Kmal is related to exopolysaccharides synthesis and biofilm formation [66].

## Regulation of *S. mutans* biofilm formation by other mechanisms

### CRISPR-associated proteins

CRISPR clusters are DNA repeats family widely present in the genome of bacteria and archaea [67]. A CRISPR locus consists of a CRISPR array bordered by various *cas* genes, which are composed of short direct repeats separated by short variable DNA sequences (called spacers) [68,69]. Two CRISPR-Cas systems exist in *S. mutans*: the CRISPR1 system (type II-A) and the CRISPR2 system (type I-C) [70].

A study found that the expression of two CRISPR/Cas locus sites in *S. mutans* is differentially regulated in *cdaA* knockout strains, which show an abnormal biofilm phenotype [23]. Zhang *et al*. found that *S*. *mutans* clinical strains with both CRIPSR systems have a stronger ability of biofilm formation [70]. Later, Tang *et al*. observed that the knockout of *cas3* reduces the ability of *S. mutans* to produce exopolysaccharides and form biofilm by decreasing the expression of *gtfB* and *gtfC* [71]. This result suggests that the CRISPR-Cas systems are involved in the regulation of *gtf*s expression and biofilm formation in *S. mutans*.

### PTS system

The PTS system consists of the nonspecific enzyme I (EI), histidine-containing the phosphocarrier protein (HPr), and sugar-specific enzyme II (EII) complexes. The EI and HPr are involved in the transport of all PTS sugars: HPr encodes *ptsH* and EI encodes *ptsI* in *S. mutans* [72]. The EII complex usually consists of three parts: EIIA, EIIB and EIIC, where EIIA and EIIB are located in the cytoplasm and EIIC is an integral membrane protein (mannose-type PTS system containing EIID) [73].

EII^Man^ is the most physiologically important PTS complex in *S. mutans*, mainly involved in the transport of glucose, mannose, galactose, glucosamine (GlcN) and N-acetylglucosamine (GlcNAc) [74,75]. This complex is made up of four domains expressed as three polypeptides in a single operon: EIIAB (*manL*), EIIC (*manM*), and EIID (*manN*). Previous studies showed that the deletion of the *manL* gene relieved CCR of several carbohydrate catabolic operons, including the *cel* operon encoding a phospho-β-glucosidase (CelA), a cellobiose-PTS EII complex [76,77], the *lac* operon encoding EII^Lac^ (*lacFE*) and proteins required for the utilization of both lactose and galactose [74]. Abranches *et al*. found that the reduced expression of the *gtfBC* promoter in the EIIAB^Man^ mutant strain is associated to the involvement of ManL in the regulation of *gtfBC* expression [78].

### Quorum-sensing signaling system

Quorum sensing is a mechanism of bacterial intercellular communication for the regulation of gene expression in response to the density of the population. It is based on the production, detection and response of extracellular signaling molecules known as autoinducers [79]. The LuxS system mediates the interspecific interactions in multi-species communities, and it is one of the widely studied quorum-sensing systems in *S. mutans* [38].

The *luxS* gene is involved in many cellular processes, and it is present in a wide range of bacteria [80]. LuxS catalyzes the formation of the autoinducer AI−2 (a furanosyl borate diester) in the methyl cycle [81]. A study found that the knockout of *luxS* in *S. mutans* suppresses *gtfD* expression [82]. In addition, its knockdown affects *S. mutans* ABC transporters and carbohydrate transport, transformation, and metabolism through the EII subunits and enzymes to influence virulence-associated traits, as revealed by a transcriptome analysis [81]. Thus, the quorum-sensing signaling system in *S. mutans* is involved in the formation of biofilms.

### Enzymes

Dextranase (Dex) is a glucanase involved in the degradation of water-soluble glucans, and includes DexA and DexB [83]. DexA breaks the α−1,6-linkage of the extracellular glucans to produce oligosaccharides, which are degraded into monosaccharides by DexB glucosidase after entering the cells [84–86]. Yang *et al*. revealed that *dexA* knockout results in an increased transcription of genes related to exopolysaccharides synthesis, including *gtfB*, *gtfD* and *ftf* [87]. In addition, the biofilms of the strains overexpressing *dexA* lack exopolysaccharides matrix and are unable to aggregate into dense and continuous microcolonies [87]. These results indicate an important role of the *dexA* gene in the formation of *S. mutans* biofilm.

YidC proteins are membrane-localized chaperone insertases that are universally conserved in all bacteria [88]. The genome of *S. mutans* contains two copies of genes encoding the YidC homologs *yidC1* and *yidC2*, and their protein sequences are 27% identical and 48% similar. The deletion of *yidC1* or *yidC2* results in a reduced synthesis of insoluble glucan at the early and mid-exponential growth phases [89]. In particular, the deletion of *yidC2* resulted in a significant reduction in biofilm biomass, evident defects in the spatial organization of the extracellular polymer matrix, and alteration in the three-dimensional biofilm structure [89]. The defective biofilm contains smaller bacterial clusters with higher cell density and less surrounding exopolysaccharides than the biofilm in the wild type [89].

The *copYAZ* is a copper-transport operon in *S. mutans* that encodes three proteins: a copper P-type ATPases (CopA), a copper-responsive repressor (CopY), and a copper chaperone (CopZ) [90]. CopY allows transcription upon copper transfer from direct binding with CopZ [90,91]. CopA is a small protein that tightly binds to copper and delivers it to CopY to positively regulate *copYAZ* [90–92]. CopZ exports the excess copper ions to the outside of the cell, causing the cell to become resistant to copper [90,91]. Garcia *et al*. found that glycosyltransferase secretion and biofilm formation are significantly reduced after *copZ* knockdown in *S. mutans* [93]. This result suggests that CopZ is a global regulator of biofilms. Further characterization of CopZ may lead to the identification of novel pathways in biofilm formation and characterization.

EzrA is an integral membrane protein consisting of an N-terminal trans-membrane (TM) spanning helix followed by an 60 kDa cytoplasmic domain [94]. It plays a key role in the regulation of cell division and in the maintenance of cell size and shape [95–97]. Xiang *et al*. investigated the role of EzrA in *S. mutans* by constructing *ezrA* in-frame deletion mutants. The results showed that the *ezrA* mutant grows slower that the wild type, with a round cell shape characterized by a shortened length and extended width [98]. Single-species cariogenic biofilm model revealed that the deletion of *ezrA* results in a defective biofilm formation with less exopolysaccharides and altered three-dimensional biofilm architecture [98].

Amyloid is a fibrous cross-β sheet quaternary structure consisting of ordered peptides or polymerized proteins [99]. *S. mutans* is an amyloid-forming organism and amyloidogenesis contributes to biofilm formation in this bacterium [100]. Three proteins are present in *S. mutans* that form amyloidogenic fibrils, including amyloidogenic adhesin SpaP, wall-associated protein A (WapA) and the secreted protein Smu.63c [101]. When *S. mutans* grows in biofilm medium containing glucose as the sole carbon source, the strains with *spaP* and *wapA* deletion produce significantly less biofilm than the wild type strain, while the deletion of *Smu.63c* results in a slight increase in biofilm production [101]. The reduction of biofilm is more significant in the *ΔspaP*/*ΔwapA* double-deletion strain and the Δ*spaP*/Δ*smu.63c*/Δ*wapA* triple-deletion strain [101]. Δ*Smu.63c*/Δ*wapA* double-deletion strain and Δ*Smu.63c*/Δ*spaP* double-deletion strain rescued the *spaP* and *wapA* deletion phenotypes, resulting in biofilm production comparable to that of the wild-type strain [101]. These results suggest that SpaP and WapA positively regulate the biofilm formation in *S. mutans*, while Smu.63c exerts a negative regulatory role.

The efflux pump is a protein localized on the cell membrane that allows the maintenance of the homeostasis of the internal environment in the microorganisms by excreting toxic substances, such as antimicrobial drugs, metabolites and signal molecules of the quorum sensing [102]. LmrB is a putative efflux pump in *S. mutans*, and its inactivation results in the structural changes of the biofilm, increased production of exopolysaccharides and increased transcription of exopolysaccharides-related genes [103]. A number of genes involved in sugar uptake and metabolism are up-regulated, including sugar metabolism associated *glg* operons and *msmREFGK* transporter, as demonstrated by the transcriptome analysis [104].

The *smu.833* gene encodes a hypothetical glycosyltransferase highly conserved among the group of mutans streptococci but absent in other oral commensals [105]. The protein encoded by *smu.833* has two transmembrane helices and is highly homologous to proteins involved in cell wall biogenesis and the response to cellular stress [105]. Rainey *et al*. found that *smu.833* regulates the dynamic interaction between glucan and extracellular DNA (eDNA) of *S. mutans* [105]. Moreover, the loss of *smu.833* resulted in a significant decrease in the expression of Gtfs and a concurrent reduction in glucan matrix [105].

The streptococcal pleiotropic regulator of virulence (SprV) is a small hypothetical protein that is encoded by *smu.2137* in *S. mutans*. The deletion of SprV leads to an impaired biofilm formation and various virulence-related functions in *S. mutans* [106]. Transcriptome sequencing revealed that *gtfB, gbpA, gbpB*, and *gbpC*, which are genes related to biofilm formation, are downregulated by 2.6-, 2.4-, 1.7-, and 2.6-fold, respectively [106].

Recombinase A (RecA) is related to the SOS-response in *S. mutans*. The RecA-deficient mutant strain possesses a lower acid tolerance and produces a biofilm with a lower density than the wild type [107]. Glutamate racemase (MurI) is an essential enzyme for the biosynthesis of peptidoglycan. *murI* deficiency in *S. mutans* weakens the ability to form the biofilm and virulence factors. The expression of *comE*, *comD*, *gtfB* and *gtfC*, genes related to biofilm formation are down-regulated 8-, 43-, 85- and 298-fold, as revealed by qRT-PCR [108].

### Small-molecule metabolites

Small molecules are often encoded by biosynthetic gene clusters (BGCs) and are the primary means of communication in the microbial world. They are involved in various microbial–microbial and host-microbial interactions, including antimicrobial activity, bacterial signaling, immunomodulation, biofilm formation, host colonization, nutrient clearance, and stress protection [109]. The genomic analysis of *S. mutans* isolates revealed that it collectively harbors a plethora of BGCs for polyketide/non-ribosomal peptide biosynthesis [110].

The polyketide/nonribosomal peptide biosynthetic gene cluster (*mufD*, *mufE*, *mufF*, *mufG*) was first identified in *S. mutans* strains isolated from dental plaque [111]. Zhong *et al*. found that the *muf* gene cluster synthesizes the small-molecule mutanofactin−697, as demonstrared by liquid chromatography – high-resolution mass spectrometry (LC – HRMS) [111]. Further mode-of-action studies showed that mutanofactin−697 binds to *S. mutans* and extracellular DNA, increases bacterial hydrophobicity and promotes bacterial adhesion and subsequent biofilm formation (Table 1) [111].

**Table 1.** Summary of genes regulating biofilm formation in S. mutans.

Gene No.	Gene Name	Inhibits or promotes exopolysaccharides synthesis and biofilm formation	References
**Regulation of *S. mutans* biofilm formation through upstream signals**			
*cdaA*	*Smu.1428c*	Cheng *et al*. found that deletion of *cdaA* promoted exopolysaccharides synthesis and biofilm formation in *S. mutans*, while Peng *et al*. found the opposite.	[23,24]
*pdeA*	*Smu.2140c*	Deletion of *pedA* promoted exopolysaccharides synthesis and biofilm formation in *S. mutans.*	[25]
*cabPA*	*Smu.1562*	-	[25]
*cabPB*	*Smu.1708*	-	[25]
*yqeK*	*Smu.1798c*	Deletion of *yqeK* inhibited exopolysaccharides synthesis and biofilm formation in *S. mutans.*	[30]
**Regulation of *S. mutans* biofilm formation at transcriptional level**			
*epsR*	*Smu.124*	Deletion of *epsR* in *S. mutans* resulted in increased water-insoluble exopolysaccharides production, and upregulated GtfB protein content and activity.	[31]
*rcrR*	*Smu.921*	Deletion of *rcrR* gene in *S. mutans* caused decreased biofilm formation ability.	[32]
*stsR*	*Smu.1193*	Deletion of *stsR* gene in *S. mutans* caused a decrease in both the formation of biofilm and the production of exopolysaccharides at early stage.	[33]
*ahrC*	*Smu.584*	Overexpression of *ahrC* in *S. mutans* showed decreased biofilm biomass, reduced production of water-insoluble exopolysaccharides.	[34]
*fruR*	*Smu.870*	Loss of *fruR* in *S. mutans* resulted in a slight reduction in biofilm mass when grown on BMFS (a biofilm medium supplemented with 18 mM fructose).	[35]
*pdxR*	*smu.864*	Deletion of *pdxR* in *S. mutans* formed significantly fewer biofilms.	[36]
*vicR*	*Smu.1517*	VicR positively regulates the expression of *gtfB*.	[39,40]
*covR*	*Smu.1924*	CovR negatively regulates the expression of the *gtfB* and *gtfC* genes by directly binding to the promoter region	[41]
*vicK*	*Smu.1516*	Deletion of *vicK* gene suppressed biofilm formation as well as exopolysaccharides production.	[45]
*vicX*	*Smu.1515*	-	[128]
**Regulation of *S. mutans* biofilm formation at post-transcriptional level**			
sRNA0426	-	sRNA0426 expression was positively correlated with exopolysaccharides production of *S. mutans.*	[52]
*rnc*	*Smu.1514*	Deletion of *rnc* inhibited biofilm formation in *S. mutans.*	[55,56]
AS vicR	-	Overexpression of AS vicR led to a reduction in biofilm formation.	[58,59]
Regulation of *S. mutans* biofilm formation at post-translation level			
*actG*	*Smu.1253c*	ActG could negatively regulate the water-insoluble exopolysaccharides and biofilm formation in *S. mutans.*	[65]
**Regulation of *S. mutans* biofilm formation by others mechanisms**			
*cas3*	*Smu.1764c*	Deletion of *cas3* in *S. mutans* led to decreased biofilm biomass and exopolysaccharides production.	[71]
*manL*	*Smu.1877*	ManL positively regulate the expression of *gtfBC*.	[78]
*luxS*	*Smu.474*	Deletion of *luxS* caused a decrease in biofilm mass.	[81,82]
*dexA*	*Smu.2042*	Deletion of *dexA* resulted in increased transcription of exopolysaccharides synthesis-related genes, including *gtfB*, *gtfD*.	[87]
*yidC1*	*Smu.337*	Deletion of *yidC1* resulted in less insoluble glucan synthesis but produced more soluble glucans, especially at early and mid-exponential-growth phases.	[89]
*yidC2*	*Smu.1727*	Deletion of *yidC2* resulted in a significant reduction in biofilm biomass and pronounced defects in the spatial organization of the extracellular polymer matrix.	[89]
*copY*	*Smu.424*	-	-
*copA*	*Smu.426*	-	-
*copZ*	*Smu.427*	Deletion of *copZ* in *S. mutans* led to a significant reduction in biofilm biomass.	[90,92,93]
*ezrA*	*Smu.1276c*	Deletion of *ezrA* resulted in defective biofilm formation with less extracellular polysaccharides and altered three-dimensional biofilm architecture.	[98]
-	*Smu.63*	Deletion of *Smu.63* led to increased sucrose-independent biofilm formation.	[101]
-	*Smu.833*	Deletion of *Smu.833* decreases glucan and increases eDNA.	[105]
*sprV*	*Smu.2137*	Deletion of *sprV* inhibited exopolysaccharides synthesis and biofilm formation in *S. mutans.*	[106]
*recA*	*Smu.2085*	Deletion of *recA* in *S. mutans* produced lower density biofilm.	[107]

## The strategies for biofilm inhibition and caries prevention

Although caries is a multi-microbial disease, selective targeting of *S. mutans* has been considered to be an appropriate approach for the prevention of dental caries [112]. This is mainly because the synthesis of exopolysaccharides from sucrose by *S. mutans* is important to the development of cariogenic biofilms. Thus, targeted inhibition of *S. mutans* biofilm may be a viable approach to impede the progression of caries without disrupting the oral microbiome associated with health.

Gtfs secreted by *S. mutans* can synthesize exopolysaccharides using sucrose [113,114]. Therefore, targeting Gtfs could selectively prevent the synthesis of exopolysaccharides, the formation of biofilm and impair the *S. mutans* virulence, which do not threaten the microecological balance in the oral cavity. Screening for compounds that inhibit Gtfs could have a significant effect on biofilm inhibition. Fluoride can inhibit the production and secretion of *S. mutans* Gtfs [115]. Some sucrose structural analogs (including 6-deoxysucrose, 6-thiosucrose, 4,6-dideoxysucrose, sucralose, 4-deoxysucrose, 4-chloro−4-deoxygalactosucrose, 6,6’-dithiodisucrose) and natural products (including Tris, green mate, roasted mate, 7-epiclusianone, cranberry juice, quercetin, polyphenols) could also inhibit Gtfs activities [116–123]. Because the specificity of these compounds is largely unknown, their access to clinical applications remains limited.

The upstream regulatory genes, including TCS and quorum sensing system, regulate *S. mutans* biofilm formation by affecting the promoter activity of *gtf*s and subsequent expression of Gtfs. Therefore, these upstream regulatory systems may be potential targets, which can be used to develop therapeutic agents against dental caries. A number of inhibitors targeting upstream regulatory pathways have been identified. WalKHK, a TCS inhibitor from *B. subtilis*, inhibits the *in vitro* autophosphorylation of VicK in *S. mutans* [124]. The cell extract from Tenacibaculum sp. 20J interfered specifically with the AI−2 quorum sensing system and reduced biofilm formation of *S. mutans* [125]. Although the expression of *gtf*s is well regulated, the exact mechanism of regulation is still not fully understood. If these regulatory genes also affect the expression of other genes (e.g. mutacins), then the strategies of targeting upstream regulatory genes may affect the oral microecological homeostasis.

Second messenger c-di-AMP and Ap4A regulate *S. mutans* biofilms formation. Inhibitors targeting synthetase/hydrolase or receptor proteins can affect the intracellular levels or signaling transduction of second messengers and inhibit biofilm formation. Currently, most c-di-AMP inhibitors target PDE and DisA [126]. For example, three inhibitors of *Bacillus subtilis* DisA, bromophenol-TH, suramin, and the tea polyphenol theaflavin digallate, were identified [126]. Stress alarmone ppGpp could competitively inhibit *B. subtilis* GdpP (PDE) [127]. However, inhibitors targeting *S. mutans* DAC/PDE still need further investigation. The mechanisms of action of these inhibitors and whether there might be an impact on the other flora in the oral cavity are still unclear.

## Conclusions

*S. mutans* is the core microorganism causing human dental caries due to its high ability of biofilm formation and cariogenicity. The regulatory network of biofilm formation mainly includes upstream signals composed of c-di-AMP and Ap4A, regulating *gtf*s gene expression by CRISPR-Cas, transcription factor, two-component systems, small RNA, PTS systems, and modulating glucosyltransferases activity by acetylation, malonylation. The specific inhibition of *S. mutans* biofilm modulates the oral microecological balance. Therefore, targeting the systems that regulate the biofilm formation might provide potential strategies for the ecological prevention and treatment of caries.
